# The development of the conversation skills assessment tool

**DOI:** 10.1177/23969415231196063

**Published:** 2023-08-24

**Authors:** Yurgos Politis, Ian Clemente, Zihyun Lim, Connie Sung

**Affiliations:** Yehuda Elkana Center for Teaching, Learning and Higher Education Research, 47797 Central European University, Austria; Department of Counselling, Educational Psychology & Special Education (CEPSE), 3078 Michigan State University, USA; Austin, Texas, USA; Department of Counselling, Educational Psychology & Special Education (CEPSE), 3078 Michigan State University, USA

**Keywords:** Conversation, autistic, virtual world, communication and language, autism

## Abstract

**Background and aims:**

Having a conversation with someone or even more within a group of people is complex. We are never taught at school how to do it, which implies we consider having a conversation as something simple and straightforward. Instead, we just learn from observing others. Some people are great conversationalists – it comes naturally to them – while others struggle. Some people may not fully understand how the process works, how turn-taking happens, don’t understand visual cues such as body language and facial expressions, and fail to comprehend that some topics may be appropriate or inappropriate. This can be the case for both neurotypical and neurodivergent people. The Conversation skills Assessment Tool has been developed in this first instance to help in assessing and examining conversation skills in an intervention with young autistic adults on a virtual platform (a virtual world). This paper will present the evolution of the new measure through the exploratory phase, the development phase and finally a detailed account of the inter-rater reliability process.

**Methods:**

The intervention associated with this study was carried out though a multiple baseline design with 3 autistic participants (in their early 20 s) and took place over 4 phases (15–17 sessions). The sessions involved semi-structured conversations in face-to-face (phases 1 and 4) and virtual (phases 2 and 3) settings and were videotaped with the participants’ consent. Twelve of those were used by this study in the development process through iterative inter-rater reliability stages between two coding teams.

**Results:**

Evaluation of the Conversation skills Assessment Tool tool revealed the potential benefit of implementing interventions with measures that more objectively and concretely (e.g., by noting frequencies) assess observable behaviours that are associated with having positive conversations with others. Beyond this, it is anticipated that Conversation skills Assessment Tool can emerge as a tool capable of not only accounting for the environment an interaction takes place in (e.g., professional, casual), but also offers beneficial feedback for both autistic students and other populations (e.g., young children, English language learners).

**Conclusions:**

This measure has the potential to offer quantifiable and trackable guidance to people who have difficulties conversing. The authors do not wish to perpetuate an ableist social construct of what is a ‘good’ conversation, nor do they suggest that conversation skills training is useful solely for people with communication and/or socialization difficulties. Rather, they hope that Conversation skills Assessment Tool can be adopted more broadly to give both neurotypical and neurodivergent people a better understanding of how to communicate more effectively with others, while also becoming more aware and accepting of differing conversational styles.

**Implications:**

Because of its ability to track (or self-monitor) one's development of conversational skills over time, Conversation skills Assessment Tool could serve as an educative tool in early childhood education. It can be used by occupational/speech therapists and other professionals and also used to self-monitor one's development of conversational skills. Conversation skills Assessment Tool was developed to assess conversation skills on a one-to-one basis; therefore, another iteration of Conversation skills Assessment Tool would have to look at group conversations.

## Introduction

There has been a documented gap between autism research and practical application (Odom et al., 2020). Dearing et al. (2018) have categorized efforts to close that gap as diffusion when the onus is on practitioners to find an appropriate and useful intervention; dissemination, when researchers have an active role in communicating about their intervention and its outcomes to practitioners and the wider public; and implementation, when researchers – in addition to disseminating and depending on a certain level of diffusion – create supports and activities that will enhance the practitioners’ capability of using an intervention effectively.

This paper is part of a series of manuscripts reporting on a Marie Curie Fellowship project that developed an intervention on conversation skills for autistic adults through the use of a virtual world (VW). The current article presents the overall process involved in the creation of this new tool, the Conversation skills Assessment Tool (ChAT). It will describe the evolution of the new measure through the exploratory phase, the development phase and finally present a detailed account of the inter-rater reliability process. While recognizing the limitations of ChAT (which will be discussed in detail here), the authors feel that practitioners using it in their practice will help improve and expand it further.

## Background

### Neurodiversity and communication/conversation

Bandura's social learning theory stresses that child development happens through observation, modelling, and imitating behaviours and emotional reactions of others (Bandura, 1977), inclusive of social interactions such as conversations. Engaging in a conversation may appear natural and effortless for some, but others may struggle because it is a complex process during which conversation partners ‘must weave shared understanding from alternating independent contributions in an act of spontaneous, dynamic cocreation’ (Wohltjen & Wheatleya, 2021: 1).

Engaging in a meaningful conversation requires the coordination of a multitude of skills in order to sustain it, such as initiating a topic, maintaining on-topic discussion, turn-taking, pauses or gaps, and how to end a conversation (Heldner & Edlund, 2010; Levinson & Torreira, 2015; Mastroianni et al., 2021; Stolk et al., 2016). Studies have shown that neurodivergent populations (particularly those who are autistic) have difficulties with topic information (e.g., use of irrelevant detail, vague or unrelated responses), topic management (e.g., inappropriate topic shifts, low rates of initiations) and reciprocity (e.g., poor responsiveness, lack of follow-up questions) (Paul et al., 2009).

Other foundations of social communication include the emotional face, voice and body expressions, which enable interactions by externalizing one's internal state and intentions to others (Russell & Dols, 2017). A conversation between two or more people also involves the ability to read facial expressions, tone and inflection of voice, hold eye contact, an ability to understand the mechanics of the pacing of an interaction, not asking too many questions and being able to ‘read between the lines’; and prior research has shown that neurodivergent populations struggle with all of those abilities to some degree (Hurlbutt & Chalmers, 2004; Globerson et al., 2015; Koldewyn et al., 2013). Acquiring the skills that enable engaging in conversations could be achieved through direct teaching or other structured interventions (e.g., Leaf & Taubman, 2011). These difficulties likely stem from the open-ended nature of a conversation (van Eylen et al., 2015) and the fact that it can be rather unstructured, when autistic individuals tend to prefer repetition or routine (National Autistic Society, 2020).

While this has been the conventional way of thinking about how conversations work, a change of mindset is occurring. There is a shift in some acceptable social norms related to social interactions, such as the height of shared attention between two conversation partners occur in the first instance of making eye contact – not during a sustained period of gazes as was previously thought (Keating & Cook, 2020).

There is also a school of thought that suggests conversations, like any other type of social interaction, happen between two (or more) *individuals*, thus differences between how individuals act and behave can and should be expected. Autistic and neurotypical individuals have differences in abilities that affect how they engage in a conversation (for example, facial expressions) (ibid.) In fact, autism ‘remains a complex condition with dissenting voices’ (Morgan, 2016). Since autism exists either as a solitary condition or with other co-morbidity(ies), it appears in a spectrum of differences in traits and characteristics. There is no consensus on whether it is a disability (anecdotally some parents consider autism a disability if they feel that their child will lead an autonomous life at best, supported with assistive products and services).

However, if ability and disability are not viewed as binary options, but rather they both have some separation from what Lambert (2018) describes as the ‘calibrating abstract body’ that acts as the threshold, then what are considered appropriate, desirable, and reasonable are fluid, and depend not only on the cohorts but also on the given context.

One of the main reasons for the development of the ChAT tool is to make an effort to quantify in a more objective way parts of the conversation process. The primary function of that would not be to compare the performance of neurodivergent to neurotypical social norms, but to give both neurotypical and neurodivergent people a better understanding of how to communicate with others, and in doing so, highlighting differing conversational styles. The ChAT tool, as you will see in the development and limitations sections of this paper, has omitted some of the above-mentioned contested areas of conversation and could be adapted for a range of neurodivergent and neurotypical cohorts and give voice to the fluid able/disable divide described above.

### Conversation skills training: The introduction of virtual reality/virtual worlds

There have been efforts to embed social skills training interventions in more natural settings for participants (children in most cases), such as home and school settings. All stakeholders playing a part in such interventions, including trainers, teachers, peers and parents, incorporate spontaneously occurring behaviours and reinforcement of child-initiated interactions and behaviours. This approach has led to notable improvements in behaviours (e.g., greeting, joint attention), but more importantly, it has done so in a manner that has facilitated increased generalization of research findings and maintenance of positive effects after the intervention (Rogers, 2000).

Techniques that have empirically emerged as effective for teaching conversational skills to children – in particular autistic adolescents – include script fading (Krantz & McClannahan, 1998), video modelling (Bellini & Akullian, 2007), and peer-mediated techniques (Kyparissos, 1996). However, recent technological advancements, including digital technologies like virtual reality (VR), have added new tools to skills training interventions for neurodivergent populations. Communication skills training in VW environments has helped autistic adolescents show improvement in initiating a conversation in VR settings (e.g., café training; Mitchell et al., 2007), and can make conversations easy, structured, and inclusive (e.g., text-chat systems; Newbutt, 2013). Autistic individuals regard VR environments as engaging and immersive, offering the opportunity to test their social skills with a sense of safety (Smith et al., 2014; Smith et al., 2015).

The tool presented in this paper is linked to a VW-based (HIVE-RD ‘R&D.Construct’ platform) conversation skills training intervention **(**the Marie Curie project described in the **‘**Marie Curie project**’** section**)** designed for autistic people that incorporated a multiple baseline design approach. It involves training across four stages (face-to-face and virtual) and varying scripted topics of conversation that differed for each session (Politis et al., 2019), thereby making finding a connection with subjects’ preferred forms of interaction and areas of interest more likely.

Building off this individualized focus, conversational difficulties can vary widely across subjects, requiring personalized interventions to address specific elements of a conversation (for example, Fey, 1986). Thus, the intervention was designed in a way that is adaptable to meet individual needs and preferences. More specifically, the design process involved a Participatory Design methodology, where people on the spectrum were involved in every stage of the development of the VW, from inception to stress-testing and feedback sessions (Politis et al., 2017). The VW was a three-dimensional fantasy space, which was accessed on a computer screen. Finally, social skills training studies have predominately focused on children in prior research, with very few studies focusing on providing such interventions for adults (for example, Lalli et al., 1991; Mesibov, 1984, for exceptions). Therefore, our study participants were young adults who had just finished school or were studying for a higher education degree, with the conversation training being aimed at equipping them with the skills necessary to facilitate their transition from education/training to autonomous or independent living.

### Marie Curie project

The first main stage of the project was the development of the VW through a Participatory Design methodology. This was an iterative process, which involved software stress test sessions with 30 users, sixteen of which were autistic, since the user-base of the HIVE-RD ‘R&D.Construct’ platform is very diverse. The testers conducted a pre-prepared sequence of tests and reported on anomalies or difficult features. This process led to significant improvements in performance (detailed description of this process presented in this paper: Politis et al., 2017).

The second main stage of the project was the development of the intervention material for use in the VW. This involved extensive research of the literature, but more so, freely available resources, tools, and training manuals. While training approaches for neurotypical people leaned towards how-to-guides (with animations), such as identifying good conversation topics (e.g., conversation starters) and ways to maintain a conversation, training approaches for neurodivergent individuals (e.g., autistic) focused on visual strategies on turn-taking and social stories about how to respond to questions.

The combined above list of topics was subsequently augmented by a set of topics from the Conversation Skills Rating Scale (CSRS) that were deemed to be feasibly observed in both the virtual and real world environments of the training and could be measured in a more objective way (not an expression of views/feelings in a Likert scale). These included:

- Speaking about partner (involvement of partner as a topic of conversation).
- Maintenance of topics and follow-up comments.
- Interruption of partner speaking turns.
- Use of time speaking relative to partner.
- Speaking rate (neither too slow nor too fast).
- Encouragements or agreements (encouragement of partner to talk).

The training material developed for the VW (powerpoints, videos and trialling a chatbot) consisted of six sessions and covered the following categories:

1. What is conversation and how does it work?
2. Why is conversation useful? And approaching someone to have a conversation.
3. Starting a conversation and why are topics appropriate and inappropriate?
4. Finding common interests.
5. Taking turns and answering questions.
6. Switching topics and ending a conversation.

The developed material was tested, through feedback sessions, with six autistic individuals and two practitioners. The feedback centred around ‘the visual presentation, pacing and organization of the content’ (Politis et al., 2017, p. 9). Delivering the training in three formats (i.e., PowerPoints, videos and chatbot) was seen favourably since the participants ‘felt that PowerPoints would represent teaching, videos would represent showing and chatbot would represent practicing’ (Politis et al., 2019, p. 7). Interestingly, the participants focused more ‘on design elements (narrative, sounds, characters, etc.), and somewhat neglected the educational content (the training materials in all three formats)’ (Politis et al., 2017, p. 10). The training material was revised based on the feedback.

The dissemination phase of the project included the afore-mentioned series of articles and conference presentations. The implementation phase involved the development of an assessment tool for conversation skills that was a better fit for the project. The CSRS was initially identified as an option for the assessment purposes of the intervention; however, this paper will explain why it was deemed necessary to initially try to adapt the CCRS, and how that led to a new tool altogether.

## Intervention methodology of the Marie Curie project

A multiple baseline design (MBD) approach was deemed the most appropriate methodology for the intervention. The three participants were young autistic adults aged 18–23 (1 female and 2 male), who were making the transition from school to further/higher education on employment. One participant was attending a university engineering course, another was attending an electronics community college course, while the third had just finished school and was working while he was considering his options. The intervention involved the researcher having conversations with each participant separately in both the real world (RW – the physical world) and a VW (a computer-generated environment, a two-dimensional world on laptop screen). The intervention took place over four phases. In Phases 1 and 4, the conversation would be in person (in RW); in Phase 2 the conversation would happen virtually (in the VW); in Phase 3, training would take place on different elements of a conversation in the VW, followed by the conversation

The conversations were semi-scripted for the first three phases in the sense that each session had a specific topic and there were five questions (question sets) that the researcher would try to ask to all participants so that there was a common base of interaction for all three participants. The topics varied, and included for instance school life, likes/dislikes, subjects, teachers, hobbies (Phase 1), and TV, films, music, travel, animals, computers (in Phases 2 and 3). The rationale for the variety of topics was that participants would have to face topics they potentially liked or disliked, felt comfortable and uncomfortable with. The question sets were used in the same order for all participants. Phase 4 was an open conversation covering topics discussed in the previous sessions. The sessions would last between 10 and 30 min.

The list below is an example of a script.

Session 8:

1. How often do you use a computer/tablet?
2. How good are your computer skills?
3. What do you use it mainly for?
4. How useful is the internet for you?
5. How safe do you feel when you are on the internet?

The participants naturally exhibited a range of interest levels with the different topics, however, they engaged satisfactorily with all of them. In the event, they would not have been able or willing to take part in a conversation about a certain topic, a new choice would have been made from a bank of pre-selected topics.

The participants were scheduled to have 4–5 sessions in Phase 1, 3 sessions in Phase 2, 6 sessions in Phase 3, and 3–4 sessions in Phase 4 (a total of 51 sessions). Participants did miss session(s) due to illness or having to deal with urgent matters, and two sessions suffered from significant technical issues, thus making the actual total 45 sessions (12 in Phase 1; 8 in Phase 2; 17 in Phase 3; 8 in Phase 4). All sessions were videotaped with the consent of the participants. The inter-rater reliability process is subsequently described and involved 12 videos, accounting for 27% of the total videos. The process resulted in Cohen's Kappa scores ranging from 0.61–1 for the seven items of the ChAT measure.

## Assessing conversation skills: The case for a new measure

When contemplating the assessment of the conversations collected through the four phases of the intervention on videos, we considered the conversation analysis (CA) methodology because it focuses on data that is naturally occurring (Potter, 2004) and the themes explored are ordinary such as everyday exchanges and stories (Paltridge, 2006). However, CA did not fit well with the chosen MBD approach because of its lack of experimental control (Hoey & Kendrick, 2017), since the objective of the MBD approach is to track personal growth. More importantly, though CA is considered difficult to comprehend and requires training since it is specialized, and the transcription is also perceived as being difficult and time-intensive (Lavin-Loucks, 2005). That would not have been an optimal choice for practitioners who are the target audience for adopting the intervention.

The authors then examined the suitability of the CSRS. The CSRS's purpose is to assess conversational competence in interpersonal settings and has been developed as a measure that is aimed at producing testable predictions for validation purposes (for example, Spitzberg, 2003). While the original intentions of the authors were to adapt the CSRS tool to meet the needs of the broader project, during this process, the authors realized they were in fact developing a new tool. The CSRS is generally well-received due to its practicality, efficiency, and reliability in assessing interpersonal communication skills (Spitzberg & Adams, 1995). However, the authors needed a measure that produced reliable results across multiple observers and different contexts (e.g., gender-based, cultural – the team consisted of members from north America, Asia, and Europe); an observational measure was needed that could be used to assess the observable behaviours associated with having a conversation not only quantitatively (i.e., by noting frequencies), but also qualitatively.

With that in mind, the authors identified six training areas that needed to be addressed in the first version of this new assessment tool: (a) Attending to the conversation; (b) common interests/switching topics; (c) turn-taking; (d) appropriate/inappropriate topics; (e) sustaining a conversation and (f) voice volume. They then tried to further unitize some of those conversation-related skills by adding levels of abstraction (a point previously raised when critiquing the CSRS), while also addressing concerns about assessing other relevant skills, such as effective listening (Daly, 1994). More specifically, the authors added an item called ‘gives appropriate answers’ and tweaked the ‘asking questions’ item to ‘asks appropriate questions’. There is precedent of such adaptations being successful in the past. For instance, Babcock et al. (1993) used a briefer, more constrained measure with conceptual similarities to the CSRS when assessing married couples’ interactions.

In the following section, the authors will describe the phases they undertook in the adaptation process of a new assessment tool. The starting point was the consideration of the CSRS to identify the elements of a conversation that would be measurable when reviewing videos from an intervention centred on conversation skills, followed by outlining the items that would comprise the adapted measure, and lastly forming a working group that would further develop the items based on initial feedback. The process described below lasted from early 2017 until early 2019.

## Results

### Development process of ChAT

#### Exploratory phase

**Figure 1.**
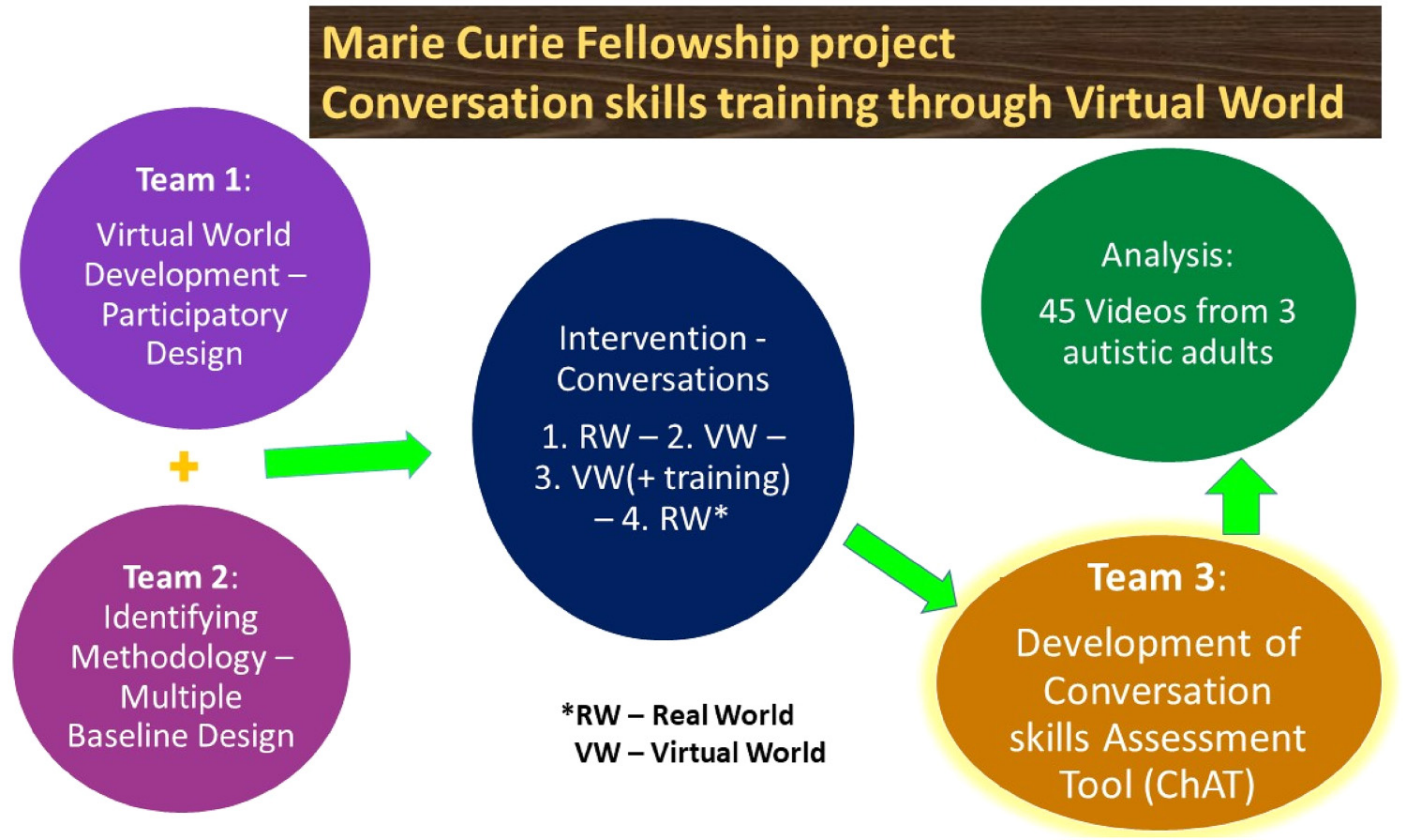
The overview of the project for which the Conversation skills Assessment Tool (ChAT) measure has been developed.

The overview of the project for which the assessment tool for conversation skills is going to be used is presented in Figure 1. A team worked on identifying the most appropriate methodology for the deployment of the intervention (Team 2), while Team 1 adapted and piloted an existing VW to be used for the intervention (Politis et al., 2017; Politis et al., 2019). The next step involved the identification of a suitable assessment tool, with which Team 3 was tasked.

As a starting point, the decision was made to adapt the 25-item CSRS by identifying specific items that could be potentially observable in the videos of the intervention sessions included in the aforementioned conversation skills training. The whole team's initial discussion focused on items that could not be (or at least not properly) observed in the VW in its current state. The discussions then focused on the fact that some CSRS items had an ableist perspective, which the team need to consider. The team opted for a consensus-building process through discussions, which were open and offered the opportunity for different perspectives to be aired, and for members to disagree in a safe, non-judgemental space (Butler & Rothstein, 2004). This process caused the removal of some items, e.g.,:

Posture (neither too closed/formal nor too open/informal)

Lean toward partner (neither too forward nor too far back)

Shaking or nervous twitches (aren’t noticeable or distracting)

That reduced the items to an initial group of 14, which were outlined in a first draft of the ChAT measure. That draft was shared with peers who had varying levels of experience and expertise in developing and using similar measures. They provided feedback on how to phrase/present items with more depth, while also discussing ways of improving their robustness in terms of detailing what is measured by each item. Consequently, the development team (i.e., the authors of this paper) was formed in mid-2017 to address that feedback. The 14 items were ultimately allocated into six training areas (Note: ‘shows continued interest in topic’ was overlapping between two training areas) and categorized as either primary or secondary outcomes.

Subsequent group discussions dealt with issues like finding ways to avoid floor/ceiling effects and trying to crystalize definitions of what constituted observable behaviours (for example, what counted as a short, medium or long response). During this process, four of the five secondary outcome items were dropped (only keeping the item ‘communicates thoughts and feelings’). The reasons for this were manifold. To give some context, all intervention sessions were videotaped for coding purposes, to assess the effectiveness of the intervention. During the Real World sessions (when the researcher and subject interacted face to face), the camera was always set on the right-hand side of the researcher (at just above head height and at a distance of roughly 2 metres away) and facing the participant. In the VW sessions (when they interacted online), the camera was placed in front of the participant (at head height and at roughly 1 m away) (Politis et al., 2017; Politis et al., 2019).

Because of this, several issues emerged. Firstly, the fact that there was just one camera used meant that it was not always clear whether or not the participant made eye contact with the researcher during the Real World sessions; and it was even more difficult to determine during the VW sessions, since the avatars did not have the capability for eye movement. Moreover, as was mentioned in the background section, recent research suggests eye contact is not as important to catching and holding a partner's attention as previously thought. Therefore, the item ‘keeps eye contact’ was dropped. Secondly, the avatars were unable to exhibit different expressions, and therefore the item involving ‘facial expressions’ could not be assessed either. Third, the item ‘vocal variety (tone/inflection)’ was dropped because the microphone source was at different distances between the RW and VW sessions (almost double the distance), which meant that it would be difficult to assess the tone in participants’ voices accurately. Lastly, the item ‘greeting (handshake/wave)’ was dropped for a few reasons. To begin, the researcher neglected on a few occasions to note whether or not the participant greeted him with a handshake or a wave (since these actions, or lack thereof, happened before the sessions started and thus were not on the videos); and when it did happen, it was unclear whether it was initiated by the participants. Moreover, during the VW sessions, the greeting would have happened in the ‘Real World’ setting and therefore the participant would probably not have considered it necessary to repeat it in the VW. Finally, on occasion, the participant and researcher met randomly outside the intervention room and greeted each other then, making a greeting moot during the intervention.

While the design of the VW and the set-up of the electronic equipment prevented the authors from collecting usable data for the further development of these items, future iterations of the tool could address these shortcomings and include some if not all of the excluded items.

#### Development phase

This led to the creation of the first full version of the ChAT assessment tool, which included one item rated on a pre-post session basis, six items rated during each interaction and four items rated for the whole session. Following feedback on the first draft of ChAT from colleagues, the authors worked to refine the tool. Consensus building through thorough discussions (similar to the exploratory phase) was employed here. This process produced a nine-item tool (since the item ‘shows continued interest in topic’ appeared in two training areas), which is shown in further detail in Table 1.

**Table 1.** Outline of second version of ChAT.

Training areas	Primary outcomes
1. Attending to the conversation	Shows continued interest in topic Shows interest for the other person in the conversation
2. Common interests/switching topics	Stays on topic
3. Turn taking	Shows continued interest in topic Allows the other person to be part of the conversation
4. Appropriate/inappropriate topics	Asks appropriate questions Gives appropriate answers
5. Sustaining a conversation	Elaborates on open-ended questions Reasonable pace
6. Voice volume	Appropriate volume level

ChAT: Conversation skills Assessment Tool.

The completion of the second version of ChAT was followed by the development of the codebook for using the tool. Two teams were created: Team 1 served as the main rater and Team 2 (consisting of two raters) would provide the level of inter-rater reliability (i.e., forming what will be referred to from now on as ‘rating teams’). One member of Team 2 focused on verbal items, whereas the other focused on nonverbal items. The coding process was tested with a trial video, which led to further tool revisions – specifically, the re-wording of four of the items, which are presented in Table 2.

**Table 2.** Description of pre-coding changes made to wording of second version of Conversation skills Assessment Tool (ChAT).

Item	Version 1 (before revisions)	Version 2 (after revisions)
1	Shows interest for the other person in the conversation	Comments about the researcher personally
2	Gives appropriate answers	Unchanged
3	Asks appropriate questions	Asks appropriate question(s) about the topic
4	Appropriate volume level	Appropriate speech volume
5	Reasonable pace	Communicates at a reasonable pace
6	Stays on topic	Unchanged
7	Shows continued interest in topic	Unchanged
8	Elaborates on open-ended questions	Unchanged
9	Allows the other person to be part of the conversation	Unchanged

Following a review of the scores from the two coding teams, it was deemed that two more items had to be dropped. More specifically, the item ‘appropriate volume level’ was deemed problematic because the camera (and thus the microphone source) was at two different distances from the participants’ faces under ‘Real World’ versus ‘Virtual World’ conditions. Specifically, the camera was almost twice the distance from participants during ‘Real World’ interactions, making it impossible to fairly compare the voice volume between the two settings. Moreover, the item ‘shows continued interest in topic’ was dropped because scoring of the item was heavily influenced by the length of the conversation on any one topic. It transpired that a short exchange would have gotten a maximum score, whereas a lengthier conversation (where the participant was often more engaged and interested in the topic) might not have gotten as high of a score. Moreover, it was difficult to define what constituted a departure from the topic because in some instances, in order for the participant to explain or give context to a term or situation, they may have appeared to digress from the original topic.

From these steps, the final version of ChAT assessment tool was born, in a manner that arguably evolved beyond adapting the CSRS and ultimately created a new type of measure, since there were few similarities remaining between the CSRS and what evolved to become the ChAT (see, Spitzberg & Adams, 1995). The items from the revised second version of the tool, which were subsequently used for the scoring of 12 videos (i.e., involving four videos from each of the three participants), are listed below:

1. Shows interest for the other person in the conversation.
2. Gives appropriate answers.
3. Asks appropriate questions.
4. Reasonable pace.
5. Shows continued interest in topic.
6. Elaborates on open ended questions.
7. Allows the other person to be part of the conversation.

It is worth repeating here that these items are based on current literature and understanding of how a conversation works, but in the context of Lambert's (2018) ‘calibrating abstract body’. What is considered appropriate, desirable, and reasonable are fluid, and these items should therefore continuously be adjusted and adapted to better meet this ever-changing understanding.

### Inter-rater reliability

The inter-rater reliability process was carried out by the two rating teams in two parts. Part 1 (December 2017 – March 2018) involved the first wave of scoring using the items listed above, while Part 2 (April 2018 – March 2019) involved revisions and re-scoring of some items following discussions between the rating teams. When there was a clear disagreement the teams justified their score based on their interpretation of the ChAT item and when that did not resolve the disagreement, additional literature was consulted to refine the language of the item and add helpful examples. The discussion thus, led to a consensus decision and subsequent revision of ChAT to reflect the changes. It is worth mentioning that the intervention involving the recorded conversations was carried out over four distinct phases. Specifically, the conversations took place in the ‘Real World’ during Phases 1 and 4 and in the ‘Virtual World’ during Phases 2 and 3. The rationale for the choice of the twelve (12) videos to assess the inter-rater reliability of ChAT was to include (a) an equal number of videos from each participant; (b) videos from both Real and VW sessions; and (c) videos from each of the first three stages of the intervention (since Stage 1 was repeated during Stage 4).

Therefore, for each participant, we chose at random one video from Phases 1 and 2 and two videos from Phase 3. That accounted for 27% of all the videos, which aligns with the approximately 25% rate commonly stated as acceptable in existing literature (Bambara et al., 2018). In each video, the two rating teams scored the five semi-scripted interactions (Politis et al., 2019) using each of the seven items of the developed ChAT measure. However, since percentage agreement does not account for that happening just by random chance, we also calculated Cohen's kappa (McHugh, 2012), which is deemed an appropriate inter-rater reliability test for nominal data (Cohen, 1960). For Cohen's kappa, a score of 0.61–0.80 is deemed substantial, and 0.81–1.00 as almost perfect.

The developed ChAT measure's seven items ultimately displayed Cohen's kappa scores ranging from 0.61 to 1:

1. Comments about the researcher personally (*k* = 0.73).
2. Gives appropriate answers (*k* = 0.61).
3. Asks appropriate question(s) about the topic (*k* = 0.62).
4. Communicates at a reasonable pace (*k* = 1.00).
5. Shows continued interests in conversation (*k* = 0.75).
6. Elaborates on open ended questions (*k* = 0.94).
7. Allows the other person to be part of the conversation (*k* = 0.64).

This process resulted in the creation of the ChAT, which is presented in Table 3.

**Table 3.** The final Conversation skills Assessment Tool (ChAT) tool.

Definition of observable behaviour					Clarifications/examples			Score
1. *Comments about the Researcher personally:* The P comments on R’ interests, opinions, personal stories. The P can also ask questions that are personal.					P: What did you do over the weekend (Personal question) P: You look tired today (Personal comment/observation)			
I1	I2	I3	I4	I5	1 – present 0 – absent			Subtotal 1
2. *Gives appropriate answers:* There is a clear connection between what the R asks/says and what the P replies or asks back.					R: What are your hobbies? (Appropriate answers) P: Fishing, hunting (Inappropriate answer) P: Annoying my brother			
I1	I2	I3	I4	I5	0 – absent 0.5 – not consistent 1 – present			Subtotal 2
3. *Asks appropriate question(s) about the topic:* P asks questions that are not too personal or potentially controversial					Appropriate: Occupation, education, interests, hobbies. Inappropriate: Age, sexual orientation, money, politics, religion.			
I1	I2	I3	I4	I5	1 – present 0 – absent			Subtotal 3
4. *Communicates at a reasonable pace:* R doesn’t need to ask the P to repeat something, unless the R has difficulty understanding the P's accent. At the same time, there aren’t long pauses after a question, that the R doesn’t need to repeat the question.					R asks a Q; P responds within 3 s; P talks at a speed that is clear and understandable *Note any consistent need to repeat a question/statement*			
Add the Number of long pauses for the whole conversation: I1 – I5					1 – Five or more 2 – Three or Four 3 – Two	4 – Once 5 – None		Subtotal 4
5*. Shows continued interest in topic:* Keeps the interaction going (i.e., asking sub-questions, making subsequent comments, laughing appropriately,). Engaged body language					Says ‘aha’ or ‘yeah’, ‘em’, ‘yes’, ‘right’, ‘sure’ or similar Nodding Looking at R, not laying back on the chair, not folding his/her arms, etc.			
I1	I2	I3	I4	I5	0 – no interest 0.5 – shows interest in part of the interaction 1 – consistent interest			Subtotal 5
*Definition of observable behaviour*					*Clarifications/ Examples*			*Score*
6. *Elaborates on open ended questions:* Does not provide a quick, brief, concise answer but rather offers a more detailed, informative and expansive response SHORT: One phrase/sentence MEDIUM: two phrases/sentences (separated by comma or full stop) LONG: Three or more phrase/sentences (separated by commas and/or full stops) *Phrase*: a few words without verb or subject *Sentence*: a verb and subject along with other words **7.**					R: What do like to do in your spare time? P: I like to *do* a lot of interesting and exciting things in my spare time, such as *play* football (1 sentence- SHORT) [*The first sentence doesn’t offer any info*] P: I like to *play* football and basketball (2 sentences - MEDIUM) [*Play basketball* is the implication] P: I like to play *football*, *soccer*, *basketball*, *tennis* and watch *Homeland*, *House of Cards*, *The Americans*, *The Good Fight*, on TV (8 Sentences- LONG) [Implication of play 4 things and watch 4 things] P: I like watching my team because they are entertaining to see play, kind to their fans and well-behaved off the court (3 sentences – LONG) [Implication: Are kind … are well-behaved..]			
I1	I2	I3	I4	I5	*First & follow-up questions in an interaction* 1 – short & short 2 – short & medium 3 – short & long		4 – medium 5 – long	Subtotal 6
7. Allows the other person to be part of the conversation: The P does not take over the conversation; he/she gives the R the chance to be equal partners in the conversation. OR does not get interrupted when trying to speak. The R's body language does not change					The R does not go quiet (not even saying ‘yeah’, ‘aha’, emm, or similar) R doesn’t start slouching, looking at the watch, looking elsewhere for more extended periods.			
Add the Number of interruptions for the whole conversation: I1 – I5 *Note* 0.5 – The participant thought the researcher finished speaking and spoke themselves 0.5 – There is a pause by both researcher and participant and then they speak together 1 – A clear interruption by the Participant					1 – four or more 2 – three 3 – twice 4 – once 5 – none			Subtotal 7
Subtotal 1 + 2 + 3 + 4 + 5 + 6 + 7 = Total score								

## Limitations

This paper has presented the development process for the ChAT, with the aim of being utilized when scoring videos collected from an intervention with young autistic adults. However, as the authors have noted here, the way in which the intervention was carried out placed certain obstacles when assessing what today are considered key areas of conversation. An item on facial expressions was not possible due to the limitations of the VW avatars, and an item involving eye contact requires the use of two cameras (at minimum) in the Real World and a more realistic avatar. An item ‘vocal variety (tone/inflection)’ could potentially be introduced to the ChAT measure in the future if the microphone source is placed at roughly the same distance from the participant during the Real World and VW sessions. Whether these types of items should be considered is debatable, since recent literature paints them as individual and subjective.

Since there were no major technical issues with the VW (i.e., the Wi-Fi did not affect the quality of the VW experience), the researcher would not need to be present when the participants did the VW sessions and therefore the item ‘greeting (handshake/wave)’ could be included with proper planning.

Finally, the item ‘shows continued interest in topic’ needs further examination for twofold reasons: Finding a way to prevent the scoring from being heavily influenced by the length of the conversation (i.e., short exchanges get a higher score); and clearly defining when departure from the topic occurs. The paradox is that for the former, a lengthier conversation would suggest that the participant is more engaged and interested in the topic; while for the latter, in order for the participant to explain or give context to a term or situation, they may sometimes appear to digress from the original topic.

## Discussion and future work

The authors have highlighted several key differences between their newly developed ChAT and the CSRS measure. Firstly, the ChAT measure seems to be better suited for semi-structured training contexts, where it can provide professionals with a measure of participants’ conversation skills ability that can help them – and the participants themselves – gauge whether or not they have achieved any personal growth. It will therefore be useful for task-oriented contexts, whereas CSRS was designed primarily for social conversational encounters. For our project, each session had a specific topic and there were five questions that the researcher would try to ask all participants. The topics covered a wide range of topics and they aligned somewhat with the participants interests, judged from the interactions with them during their onboarding to the intervention.

Secondly, while the scoring of CSRS may not require any training (although it is recommended for consistency across observers), a codebook has been developed for ChAT with instructions revised on multiple occasions during its development process. Thirdly, while CSRS is a self- or other-reported measure that can be completed in five to seven minutes, ChAT requires taking a video recording of a given interaction to complete an assessment, thus potentially making the scoring of the items significantly more time-consuming. However, it is hoped that this will also make the scoring distribution for ChAT more evenly balanced, in a manner that could compensate for CSRS's ceiling effects – namely, the tendency for its scores to be on the higher end due to the large majority of people being scored as relatively competent rather than incompetent.

Because of its ability to track (or self-monitor) one's development of conversational skills over time, ChAT could also serve as a useful educative tool in early childhood or for those learning the English language (i.e., by highlighting key elements of successful conversations). But even further, it could also be invaluable for offering quantifiable and trackable guidance for people who have significant difficulties in having positive conversations with others. However, the authors do not wish to perpetuate an ableist social construct of what is a ‘good’ conversation, nor do they suggest that conversation skills training is necessary solely for people with communication and/or socialization differences. Rather, they hope that ChAT can be adopted more broadly to give both neurotypical and neurodivergent people a better understanding of how to communicate more effectively with others, while also enabling them to become more aware and accepting of differing conversational styles.

Because of these considerations, the authors are fully aware that ChAT will need to undergo rigorous testing in the coming years. With this in mind, they intend on making the assessment tool available to the public through open access, so that independent research teams can utilize it in their own projects and offer feedback on ways it could be improved. This could happen through their offering suggestions for re-wording some of the coding criteria or getting feedback from participants as to how they feel some Items can become more relevant to their lived experiences. Moreover, it would also be useful if other professionals (e.g., speech and language experts, occupational therapists) can test ChAT with a wide range of age groups, which could highlight potential customizations and adaptations to help people address social scenarios encountered at different stages of life. Furthermore, its level of usefulness could then be examined across a spectrum of conditions and contexts (e.g., various intellectual and developmental disabilities, learning how to communicate in English as a second language, rehabilitating after experiencing a traumatic brain injury that have affected one's communication skills). Thus, it is hoped that ChAT could ultimately be customized with a neurodiverse population in mind, in terms of identifying adaptations of the tool that might benefit both neurotypical and neurodivergent populations.

Worth keeping in mind though that the current version of ChAT was developed to assess conversation skills on a one-to-one basis, the simplest form of social interaction. Therefore, if another iteration of the tool focuses on group settings, it must account for the different dynamics that exist between participants during such interactions. For instance, such a version of ChAT will need an item to ascertain the participant's ability to appropriately signal to an ‘outsider’ whether or not they are welcome to join the conversation. A further suggestion would be an item related to one's perception of the group dynamic, which assesses an individual's ability to determine whether or not a group of people having a conversation are open/welcoming of new conversation participants. Moreover, it would be useful to add items involving group-centred behaviours, such as joining a group interaction successfully, following the flow of the conversation without being disruptive and making themselves heard (i.e., speaking up) when they feel uncomfortable. In summary, the ChAT measure will need to be revised to take into account the multi-person aspect of such interactions.

The authors acknowledge that this is merely a starting point for this measure and that future iterations will progress and refine it further. Following an 18-month process that culminated with the development of ChAT, the authors feel that future progress cannot be achieved without outside/third-party contributions. Thus, with a focus on continuing to utilize a participatory design approach for future development of ChAT, the authors await with interest feedback, suggestions and insights offered by all relevant stakeholders (academic and non-academic alike).
